# Diagnostic Performance of ^18^F-FDG PET or PET/CT for Detection of Post-Transplant Lymphoproliferative Disorder: A Systematic Review and a Bivariate Meta-Analysis

**DOI:** 10.3390/diagnostics10020101

**Published:** 2020-02-12

**Authors:** Veronika Ballova, Barbara Muoio, Domenico Albano, Francesco Bertagna, Luca Canziani, Michele Ghielmini, Luca Ceriani, Giorgio Treglia

**Affiliations:** 1 Department of Oncology and Hematology, Cantonal Hospital Baden, CH-5404 Baden, Switzerland; veronika.ballova@ksb.ch; 2 Clinic of Medical Oncology, Oncology Institute of Southern Switzerland, Ente Ospedaliero Cantonale, CH-6500 Bellinzona, Switzerland; barbara.muoio@eoc.ch; 3 Nuclear Medicine, University of Brescia and Spedali Civili Brescia, IT-25123 Brescia, Italy; doalba87@libero.it (D.A.); francesco.bertagna@unibs.it (F.B.); 4 Department of Haematology and Oncology, Niguarda Hospital, IT-20162 Milan, Italy; luca.canziani@ospedaleniguarda.it; 5 Academic Education Research and Innovation Area, General Directorate, Ente Ospedaliero Cantonale, CH-6500 Bellinzona, Switzerland; michele.ghielmini@eoc.ch; 6 Faculty of Biomedical Sciences, Università della Svizzera Italiana (USI), CH-6900 Lugano, Switzerland; 7 Clinic of Nuclear Medicine and Molecular Imaging, Imaging Institute of Southern Switzerland, Ente Ospedaliero Cantonale, CH-6900 Lugano, Switzerland; 8 Institute of Oncology Research, CH-6500 Bellinzona, Switzerland; 9 Department of Nuclear Medicine and Molecular Imaging, University of Lausanne and Lausanne University Hospital, CH-1011 Lausanne, Switzerland

**Keywords:** PET, ^18^F-FDG, PTLD, lymphoma, post-transplant lymphoproliferative disorder, meta-analysis

## Abstract

Background: Some studies evaluated the diagnostic performance of fluorine-18-fluorodeoxyglucose (^18^F-FDG) positron emission tomography or positron emission tomography/computed tomography (PET or PET/CT) for the detection of post-transplant lymphoproliferative disorder (PTLD). As there is no clear consensus about the diagnostic accuracy of these imaging methods, we performed a meta-analysis on this topic. Methods: A comprehensive computer literature search of PubMed, Embase, and Cochrane library databases through December 2019 was performed. Pooled sensitivity, specificity, positive and negative likelihood ratios (LR+ and LR−), and diagnostic odds ratio (DOR) of ^18^F-FDG PET or PET/CT for detection of PTLD were calculated. Results: Five studies reporting data on the diagnostic performance of ^18^F-FDG PET or PET/CT in 336 transplant recipients were included in the systematic review and bivariate meta-analysis. Pooled sensitivity and specificity for detection of PTLD were 89.7% (95% confidence interval (95%CI): 84.6–93.2%) and 90.9% (95%CI: 85.9–94.3%), respectively. Pooled LR+, LR−, and DOR were 8.9 (95%CI: 5.7–14), 0.13 (95%CI: 0.08–0.2), and 70.4 (95%CI: 35.4–140), respectively. A significant heterogeneity among studies was not detected. Conclusions: Despite limited literature data, ^18^F-FDG PET or PET/CT demonstrated good diagnostic performance for the detection of PTLD, but large prospective studies are needed to strengthen these findings.

## 1. Introduction

The term post-transplant lymphoproliferative disorder (PTLD) includes a heterogeneous group of lymphoproliferative lesions with highly variable clinical and pathological features, occurring as complications after solid organ transplant (SOT) or allogeneic hematopoietic cell transplant (HCT) [1]. Taking into account the last update of the WHO classification of tumors of hematopoietic and lymphoid tissues, PTLD includes the following entities: nondestructive PTLD (previously called early PTLD), polymorphic PTLD, monomorphic PTLD (B-cell, or T/NK-cell lymphomas), and classical Hodgkin lymphoma PTLD. Monomorphic PTLD, most commonly diffuse large B-cell lymphoma, is the most frequent PTLD category [2]. It should be emphasized that, according to literature data, PTLD is characterized by a high incidence of extranodal involvement [1,2].

As PTLD may be associated with significant morbidity and mortality, a prompt and accurate diagnosis of this serious post-transplant complication is needed. The clinical diagnosis of PTLD can be challenging due to its nonspecific and highly variable presentation; on the other hand, histopathology and adequate immunophenotyping are essential to confirm the diagnosis [1].

Regarding the diagnostic imaging methods, current National Comprehensive Cancer Network (NCCN) guidelines recommend contrast-enhanced computed tomography (CECT) and/or fluorine-18 fluorodeoxyglucose positron emission tomography/computed tomography (^18^F-FDG PET/CT) as part of initial diagnostic workup for PTLD [3].

^18^F-FDG PET or PET/CT may be used to detect PTLD, due to the increased ^18^F-FDG uptake and high glycolytic metabolism of most lymphoproliferative disorders [4].

According to the recent guidelines of the American Society of Transplantation, ^18^F-FDG PET or PET/CT could provide additional useful information for staging and end-of-treatment response assessment in patients with PTLD [5], but there is no clear consensus about the diagnostic performance of these imaging methods [4]. Therefore, we aim to perform a systematic review and bivariate meta-analysis of the diagnostic performance of ^18^F-FDG PET or PET/CT for detection of PTLD to provide timely, evidence-based data in this setting.

## 2. Methods

This systematic review and meta-analysis was performed according to the “Preferred Reporting Items for a Systematic Review and Meta-Analysis of Diagnostic Test Accuracy Studies” (PRISMA-DTA statement) [6] and takes into account additional practical guidelines on systematic reviews and meta-analyses of diagnostic test accuracy in nuclear medicine [7,8].

### 2.1. Search Strategy

Three coauthors (B.M., L.Ca. and G.T.) independently performed a comprehensive computer literature search of PubMed, Embase, and Cochrane library databases to find relevant published studies on the diagnostic performance of ^18^F-FDG PET or PET/CT for detection of PTLD.

A search algorithm based on a combination of terms related to the index test (A), the target condition (B), and the outcome measure (C) was created: (A) “FDG” OR “fluorodeoxyglucose” OR “PET” OR “positron emission tomography” AND (B) “PTLD” OR “post-transplant*” OR “posttransplant*” AND (C) “sensitivity” OR “specificity.” No beginning date limit nor language restrictions were used. The literature search was updated until 31 December 2019. References of the retrieved articles were also screened to search for possible additional articles.

### 2.2. Study Selection

Studies assessing the diagnostic performance of ^18^F-FDG PET or PET/CT for detection of PTLD and reporting data about sensitivity and specificity were eligible for inclusion in the qualitative (systematic review) and quantitative analysis (meta-analysis). The exclusion criteria were: (a) articles not within the field of interest (including those about ^18^F-FDG PET or PET/CT in PTLD but not reporting data on diagnostic performance in terms of sensitivity and specificity), (b) editorials or letters, review articles, comments, conference proceedings, and (c) case reports or small case series.

Four coauthors (B.M., L.Ca., V.B., and G.T.) performed the identification of eligible records, the screening of the abstracts, the selection of the articles, and their inclusion according to the eligibility criteria. A consensus meeting held at Ente Ospedaliero Cantonale (Bellinzona, Switzerland) in January 2020 was useful to solve any disagreement among the coauthors.

### 2.3. Data Extraction

The information collected for each article included the following: name of authors, year of publication, country of origin, study design, number of patients, median age of patients and their sex ratio, type of transplanted organ, Epstein-Barr virus (EBV) status, time between transplant and PTLD diagnosis or PET acquisition, type of PET modality used, mean injected activity of ^18^F-FDG, time between ^18^F-FDG injection and PET acquisition, PET or PET/CT protocol, PET image analysis, reference standard, data on diagnostic performance of ^18^F-FDG PET or PET/CT for detection of PTLD on a per patient- or a per examination-based analysis, and type of PTLD histologically proven.

### 2.4. Quality Assessment

We have used the Quality Assessment of Diagnostic Accuracy Studies (QUADAS-2) tool for quality appraisal of the studies included in this systematic review [9]. Four domains (patient selection, index test, reference standard, and flow and timing) were evaluated in terms of risk of bias, and three domains (patient selection, index test, and reference standard) were also assessed in terms of concerns regarding applicability [9].

### 2.5. Statistical Analysis

The following metrics were obtained from the individual studies on a per patient- or per examination-based analysis: sensitivity, specificity, positive and negative likelihood ratios (LR+ and LR−), and diagnostic odds ratio (DOR) of ^18^F-FDG-PET or PET/CT for detection of PTLD. We used a bivariate random-effects model to obtain pooled sensitivity and specificity, because this statistical approach takes into account any possible correlation between sensitivity and specificity [8]. We used a random-effects model to obtain pooled LR+, LR−, and DOR. Pooled data were presented with 95% confidence interval values (95%CI) and displayed using forest plots.

Heterogeneity among studies was estimated by using the I-square index (I^2^), and a statistically significant heterogeneity was considered significant if I^2^ was greater than 50% [10]. Publication bias was assessed through the Egger’s test [11].

OpenMeta[Analyst]**^®^** statistical software (Rockville, Maryland, United States) was used for the meta-analysis.

## 3. Results

### 3.1. Literature Search

A total of 34 records were identified through the comprehensive computer literature search of the selected databases. Screening 34 abstracts, 29 records were excluded: 26 because they were not focused on the search question of this systematic review and meta-analysis, two as editorials, reviews, or letters, and one as a small case series. Lastly, five articles were selected and retrieved in full text. No additional records were found screening the references of these articles. Therefore, five studies were included in the qualitative analysis (systematic review) and in the quantitative analysis (meta-analysis) [12,13,14,15,16].

**Figure 1:**
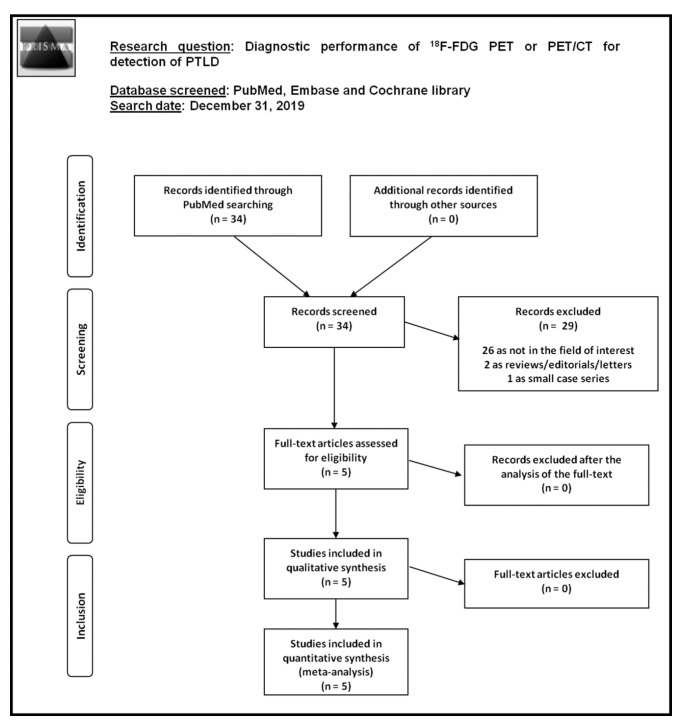
Flow chart of the search for eligible studies on the diagnostic performance of fluorine-18-fluorodeoxyglucose positron emission tomography (^18^F-FDG PET) or positron emission tomography/computed tomography (PET/CT) for detection of post-transplant lymphoproliferative disorder (PTLD).

The results of the comprehensive literature search are summarized in Figure 1.

**Figure 2:**
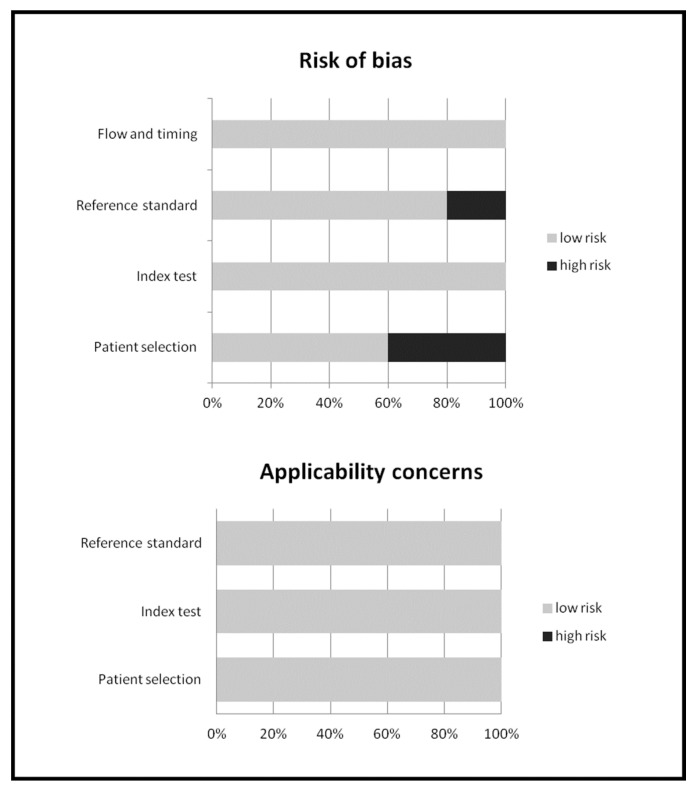
Overall quality assessment of the studies included in the systematic review according to Quality Assessment of Diagnostic Accuracy Studies (QUADAS-2) tool.

The characteristics of the studies included in the systematic review are presented in Table 1, Table 2, Table 3 and Table 4, whereas the overall quality assessment of the studies included in the meta-analysis is reported in Figure 2.

### 3.2. Qualitative Analysis (Systematic Review)

#### 3.2.1. Basic Study and Patient Characteristics

Screening the selected databases, five articles evaluating the diagnostic performance of ^18^F-FDG PET or PET/CT for detection of PTLD in 336 transplant recipients were selected (Table 1) [12,13,14,15,16]. All the selected articles were retrospective single-centre studies published from 2009 to 2019 by research groups from different countries, mainly from Europe. The median age of patients included in these studies ranged from 42 to 54 years, and the percentage of male patients (sex ratio) ranged from 55% to 87%. The type of transplanted organ was quite heterogeneous among the included studies.

#### 3.2.2. Technical Aspects

Technical details about ^18^F-FDG PET or PET/CT in the included studies are summarized in Table 2. Hybrid ^18^F-FDG PET/CT was performed in most of the cases: CT was used for PET reconstruction as attenuation map and for anatomical reference. The analysis of PET and PET/CT images was performed by using qualitative criteria (visual analysis) in all the studies. At visual analysis, all the areas of focal increased ^18^F-FDG uptake (greater than the surrounding tissue or the mediastinal blood pool) not judged as physiological activity or due to other diseases were considered to be positive for PTLD. Additional semiquantitative criteria, i.e., through the calculation of the standardized uptake values (SUV), were less frequently used. A composite reference standard including histopathology (gold standard) or clinical/biochemical/imaging data (if histopathology data were not available) was used in the included studies.

#### 3.2.3. Main Findings

In most of the included studies, a good diagnostic performance of ^18^F-FDG PET or PET/CT for detection of PTLD was reported (Table 3). The most frequent type of PTLD demonstrated by histology was monomorphic PTLD (Table 4).

False positive results of ^18^F-FDG PET or PET/CT for detection of PTLD could be divided into two main categories: other tumors also showing high ^18^F-FDG uptake, and infectious or inflammatory diseases also taking up ^18^F-FDG. False-negative results occurred when PTLD lesions were located in areas of high physiological ^18^F-FDG uptake, in cases of non-^18^F-FDG avid PTLD (such some nondestructive and polymorphic PTLD lesions), and in cases erroneously interpreted as inflammatory lesions or other tumors [12,13,14,15,16].

As recently reported by Montes de Jesus et al., the inter-observer variability in reading ^18^F-FDG PET/CT images for detection of PTLD was good. Among the parameters hypothesized to be associated with a true positive ^18^F-FDG PET/CT result for the diagnosis of PTLD, only lactate dehydrogenase was statistically significant [12].

Gheysens et al. demonstrated a high diagnostic performance of ^18^F-FDG PET/CT in detecting bone marrow involvement of PTLD, reporting a significantly higher sensitivity of ^18^F-FDG PET/CT compared to bone marrow biopsy (100% versus 17%, respectively) but similar specificity [13].

Compared to conventional imaging methods, ^18^F-FDG PET/CT may detect additional lesions in some cases of PTLD, which may lead to an upstaging of the disease [14,16]. Nevertheless, a statistically significant difference in terms of sensitivity and specificity of ^18^F-FDG PET/CT compared to CT for detection of PTLD was not demonstrated [14].

### 3.3. Quantitative Analysis (Meta-Analysis)

Five retrospective studies were selected for the bivariate meta-analysis [12,13,14,15,16].

**Figure 3:**
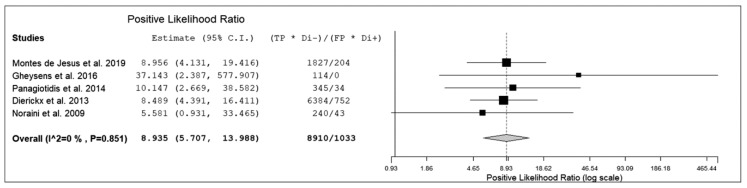
Forest plot of individual studies and pooled positive likelihood ratio of ^18^F-FDG PET or PET/CT for detection of PTLD, including 95% confidence interval.

**Figure 4:**
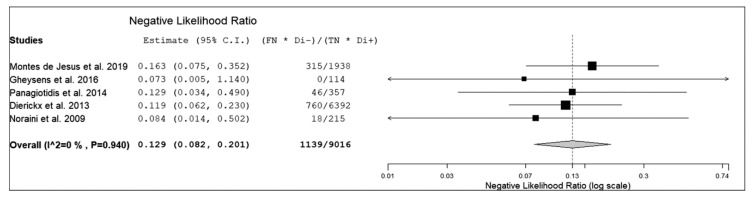
Forest plot of individual studies and pooled negative likelihood ratio of ^18^F-FDG PET or PET/CT for detection of PTLD, including 95% confidence interval.

**Figure 5:**
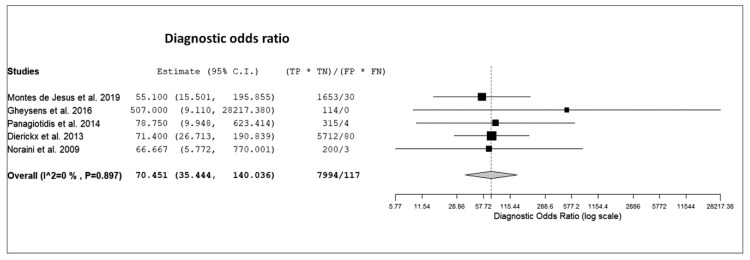
Forest plot of individual studies and pooled diagnostic odds ratio of ^18^F-FDG PET or PET/CT for detection of PTLD, including 95% confidence interval (95%CI).

The sensitivity of ^18^F-FDG PET or PET/CT for detection of PTLD in transplant recipients ranged from 85% to 100%, with a pooled estimate of 89.7% (95%CI: 84.6–93.2%). The specificity of ^18^F-FDG PET or PET/CT for detection of PTLD in transplant recipients ranged from 83% to 100% with a pooled estimate of 90.9% (95%CI: 85.9–94.3%). The pooled LR+, LR-, and DOR were 8.9 (95%CI: 5.7–14), 0.13 (95%CI: 0.08–0.2), and 70.4 (95%CI: 35.4–140), respectively (Figure 3, Figure 4 and Figure 5).

No significant statistical heterogeneity among the included studies was found for all the metrics evaluated (*I*^2^ < 50%). No significant publication bias was detected by the Egger’s test (*p* > 0.1).

Performing a sensitivity analysis, leaving out from the pooled analysis the study of Gheysens et al. because it focused only on bone marrow localizations of PTLD, the pooled sensitivity and specificity of ^18^F-FDG PET or PET/CT for detection of PTLD in transplant recipients did not change significantly, with pooled estimates of 89.1% (95%CI: 83.7–92.9%) and 89.8% (95%CI: 84.2–93.6%), respectively.

## 4. Discussion

Some studies have evaluated the diagnostic performance of ^18^F-FDG PET or PET/CT (in terms of sensitivity or specificity) for detection of PTLD in transplant recipients [12,13,14,15,16]. We have pooled data reported in the published studies through a bivariate meta-analysis to obtain more robust estimates on the diagnostic performance of ^18^F-FDG PET or PET/CT in this setting compared to the single original studies. The hierarchical methods (including the bivariate random-effects model) are considered the most appropriate tools for pooling sensitivity and specificity from multiple diagnostic test accuracy studies, because they take into account any correlation that may exist between sensitivity and specificity [8].

Overall, despite the relatively limited data available from the literature, our systematic review and bivariate meta-analysis demonstrated a good diagnostic performance of ^18^F-FDG PET or PET/CT for detection of PTLD in transplant recipients.

A recent evidence-based article reported that ^18^F-FDG PET/CT is currently the most frequently investigated imaging modality for the diagnosis and staging of PTLD in transplant recipients [17]. In this setting, ^18^F-FDG PET/CT may identify hypermetabolic foci for possible diagnostic biopsy. Moreover, ^18^F-FDG PET/CT may detect additional PTLD lesions compared to conventional imaging modalities in about one third of cases, mainly in extranodal sites, resulting in possible PTLD upstaging in some cases [14,16,17].

Nevertheless, false positive findings of ^18^F-FDG PET/CT, mainly due to inflammatory conditions, infections, or other malignancies, should be taken into account [12,13,14,15,16,17]. However, infectious diseases and tumors represent common complications in transplant recipients and their detection by ^18^F-FDG PET/CT in patients with suspicious PTLD should be considered as clinically relevant findings and not only as false positive results [18,19].

Even if the sensitivity of ^18^F-FDG PET/CT in detecting PTLD is high, possible false-negative results of this imaging method may be due to PTLD lesions located in the areas of high physiological ^18^F-FDG uptake or some cases of nondestructive and polymorphic PTLD lesions and these findings should be kept in mind by PET/CT readers [12,13,14,15,16,17].

Based on the literature data available so far, additional studies on the diagnostic performance of ^18^F-FDG PET/CT for detection of PTLD are required. Given this, prospective and multicenter studies including larger populations to better characterize the diagnostic performance of ^18^F-FDG PET/CT in the different subtypes of this heterogeneous entity are needed. In particular, a comparative analysis of diagnostic performance of ^18^F-FDG PET/CT in different patient populations (pediatric or adult patients) or different types of PTLD is recommended.

Even if outside the field of interest of our meta-analysis, it should be underlined that, beyond diagnosis and staging of PTLD, ^18^F-FDG PET/CT is emerging as a useful imaging modality to evaluate the treatment response in patients with PTLD since it alters or provides additional treatment guidance in about one third of cases [17]. Recent published data demonstrated that negative ^18^F-FDG PET/CT after treatment may identify PTLD patients with low risk of disease recurrence, due to its high negative predictive value, providing clinically relevant information [20,21].

Some limitations of our meta-analysis should be listed. As this is an emerging topic, a limited number of studies including information on the diagnostic performance of ^18^F-FDG PET or PET/CT in PTLD were available for this systematic review and meta-analysis, thus influencing the statistical power and hampering the generalization of the results. Therefore, more studies on the diagnostic performance of ^18^F-FDG PET or PET/CT in PTLD are warranted. As a composite reference standard was used in the included studies, a possible verification bias could not be excluded, but most of the lesions detected by ^18^F-FDG PET/CT were confirmed by histopathology. Furthermore, based on the available data, a selection bias could be present.

Heterogeneity among studies (i.e., due to differences among patients included, methodological aspects, and study quality) may represent a potential source of bias in a meta-analysis [8]. Nevertheless, we have not detected a statistically significant heterogeneity among the included studies in our meta-analysis. Moreover, a significant publication bias was not demonstrated in our meta-analysis.

## 5. Conclusions

Based on the available literature data, ^18^F-FDG PET and PET/CT seem to demonstrate good diagnostic performance for the detection of PTLD. The literature on this topic is still limited, and further investigations on the diagnostic performance of ^18^F-FDG PET/CT for detection of PTLD are warranted.

## Figures and Tables

**Table 1:** Basic study and patient characteristics.

Authors	Year	Country	Study design	Number of Patients Performing ^18^F-FDG PET or PET/CT	Median Age (Years)	% Male	Percentage of EBV Positivity	Transplanted Organ	Median Time Between Transplant and PTLD Diagnosis or PET Acquisition (Months)
Montes de Jesus et al. [12]	2019	Netherlands	retrospective	91	54	55%	62%	lung (44%), kidney (34%), liver (12%), HSC (4%), other (6%)	60
Gheysens et al. [13]	2016	Belgium	retrospective	25	42	60%	NR	kidney (40%), lung (40%), heart (8%), liver (8%), other (4%)	76.5
Panagiotidis et al. [14]	2014	United Kingdom	retrospective	40	52	55%	NR	liver (40%), kidney (40%), heart (10%), other (10%)	112
Dierickx et al. [15]	2013	Belgium	retrospective	150	NR	60%	56%	kidney (34%), liver (15%), lung (15%), heart (15%), HSC (15%), other (6%)	69
Noraini et al. [16]	2009	Malaysia, Italy	retrospective	30	49.5	87%	NR	liver (47%), heart (40%), kidney (10%), lung (3%)	NR

Legend: ^18^F-FDG = fluorine-18 fluorodeoxyglucose; CT = computed tomography; EBV = Epstein–Barr virus; HSC = hematopoietic stem cells; NR = not reported; PET = positron emission tomography; PTLD = post-transplant lymphoproliferative disorder.

**Table 2:** Technical aspects of the included studies.

Authors	PET Modality	Mean Injected Activity of ^18^F-FDG	Time between ^18^F-FDG Injection and PET Acquisition	PET Protocol	Image Analysis	Reference Standard
Montes de Jesus et al. [12]	PET/CT	3 MBq/kg	60 min	Images from the skull base to mid-thigh	visual	Histology or clinical/biochemical/imaging data
Gheysens et al. [13]	PET/CT	NR	60 min	Images from the skull to mid-thigh	visual	Histology or clinical/biochemical/imaging data
Panagiotidis et al. [14]	PET/CT	370 MBq	60 min	Images from the skull base to mid-thigh	visual	Histology or clinical/biochemical/imaging data
Dierickx et al. [15]	PET or PET/CT	4 x body weight (kg) + 20 MBq	60 min	Images from the skull to mid-thigh	visual and semiquantitative	Histology or clinical/biochemical/imaging data
Noraini et al. [16]	PET/CT	259-333 MBq	60 min	Images from the skull base to mid-thigh	visual and semiquantitative	Histology or clinical/biochemical/imaging data

Legend: ^18^F-FDG = fluorine-18 fluorodeoxyglucose; CT = computed tomography; NR = not reported; PET/CT = positron emission tomography/computed tomography.

**Table 3:** Diagnostic performance of ^18^F-FDG PET or PET/CT for detection of PTLD on a per patient- or a per examination-based analysis.

Authors	True Positive	False Negative	False Positive	True Negative	Sensitivity	Specificity	PPV	NPV	Accuracy
Montes de Jesus et al. [12]	29	5	6	57	85%	90%	83%	92%	89%
Gheysens et al. [13] *	6	0	0	19	100%	100%	100%	100%	100%
Panagiotidis et al. [14]	15	2	2	21	88%	91%	88%	91%	90%
Dierickx et al. [15]	84	10	8	68	89%	89%	91%	87%	89%
Noraini et al. [16]	40	3	1	5	93%	83%	98%	63%	92%

Legend: * = only assessment of bone marrow involvement; NPV = negative predictive value; PPV = positive predictive value.

**Table 4:** Type of PTLD histologically proven in the included studies.

Authors	Monomorphic PTLD	Polymorphic PTLD	Early lesions(nondestructive PTLD)	Hodgkin-like PTLD	Unclear histology
Montes de Jesus et al. [12]	70%	18%	6%	3%	3%
Gheysens et al. [13]	84%	8%	8%	0%	0%
Panagiotidis et al. [14]	93%	0%	0%	7%	0%
Dierickx et al. [15]	82%	3%	12%	3%	0%
Noraini et al. [16]	76%	21%	0%	3%	0%
